# Evaluation performance of diagnostic methods of intestinal parasitosis in school age children in Ethiopia

**DOI:** 10.1186/s13104-015-1822-4

**Published:** 2015-12-26

**Authors:** Mulat Yimer, Tadesse Hailu, Wondemagegn Mulu, Bayeh Abera

**Affiliations:** Department of Medical Microbiology, Immunology and Parasitology, College of Medicine and Health Science, Bahir Dar University, Bahir Dar, Ethiopia

**Keywords:** Wet mount, Sensitivity, NPV, Kato Katz, FEC, Ethiopia

## Abstract

**Background:**

Although the sensitivity of Wet mount technique is questionable, it is the major diagnostic technique for routine diagnosis of intestinal parasitosis in Ethiopia. Therefore, the aim of this study was the evaluation performance of diagnostic methods of intestinal parasitosis in school age children in Ethiopia.

**Methods:**

A cross sectional study was conducted from May to June 2013. Single stool sample was processed for direct, Formol ether concentration (FEC) and Kato Katz methods. The sensitivity and negative predictive value (NPV) of diagnostic tests were calculated in terms of the “Gold” standard method (the combined result of the three methods altogether).

**Results:**

A total of 422 school age children were participated in this study. The prevalence of intestinal parasites was high (74.6 %) with Kato Katz technique. The sensitivity of Wet mount, FEC and Kato Katz tests against the Gold standard test was 48.9, 63.1 and 93.7 %, respectively. Kato Katz technique revealed a better NPV 80.4 (80.1–80.6) as compared to the Wet mount (33.7 %) and FEC techniques (41.3 %).

**Conclusion:**

In this study, the Kato Katz technique outperformed the other two methods but the true values for sensitivity, specificity and diagnostic values are not known. Moreover, it is labor intensive and not easily accessible. Hence, it is preferable to use FEC technique to complement the Wet mount test.

## Background

Intestinal parasitoses are common parasitic infections in Ethiopia especially in school age children. And hence, various techniques have been used for examination of intestinal parasitic diseases [1]. However, direct wet mount, Formol ether concentration (FEC) and Kato Katz techniques have been used as a means of diagnosis for several years [2]. Despite this, the performance of immunoassays such as indirect hemagglutination assay, enzyme-linked immunosorbent assay, and dipstick dye immunoassay has recently been assessed. As a result, immunoassays have high sensitivities (>90 %) and ease of use when compared with coprological methods, but issues of specificity and the inability of antibody detection methods to distinguish between current and past infections are still of concern [3, 4].

Although other diagnostic methods such as Kato-Katz and FEC techniques are available, direct wet mount is commonly used as a diagnostic method for the diagnosis of both protozoal and helminthic infections generally in Africa and particularly in Ethiopia [5, 6]. However, low sensitivity of the direct wet mount technique has been reported in the detection of low intensity of infection [7].

The sensitivity of the FEC was higher than that of the Kato-Katz method for the diagnosis of soil transmitted helminths and *Taenia* species except *Schistosoma mansoni* [8]. The recovery efficiency of the FEC for helminthic eggs, protozoan oocysts and cysts is superior to the direct smear [9, 10]. The Kato Katz test has also superior sensitivity than the direct wet mount microscopic stool examination [11]. This shows that the use of direct wet mount as a confirmatory test will significantly increase under diagnosis of false negative test results.

The reliable diagnosis of intestinal parasitic infections requires a more rapid, easy, and sensitive method. The Wet mount method has been chosen as a routine diagnosis because it is easy to perform, had low cost and time saving as compared to other two techniques. The detection rate of parasites in a single stool examination using Wet mount method is very limited due to poor sensitivity [12].

As a result, the chance of false negative results will be high. Consequently, under diagnosis of intestinal parasitosis among patients resulted in and mislead of the physicians may take place as above. Evaluating the performance of Wet mount method against the most sensitive methods (Kato Katz and FEC) is very important to address diagnostic challenges in the area. Moreover, the available information with respect to Kato Katz and FEC in the study area is limited. Therefore, the aim of this study was the evaluation performance of diagnostic methods of intestinal parasitosis in school age children in Ethiopia.

## Methods

### Study design and area

A cross sectional study was conducted in school age children in Tach Armachiho Ethiopia from May to June 2013. The Tach Armachiho is one of the woredas in the Amhara National Regional states of Ethiopia. It is found 260 km away from Bahir Dar, the capital city of the region. The prevalence of intestinal parasites is high in the region especially in remote area like Tach Armachiho due to low sanitation and hygiene. As a result, we were interested to conduct this research in Tach Armachiho area.

### Sample collection procedure

A total of 422 primary school age children were selected as study participants. Of these, 135 (31.9 %) were males and 287 (68 %) were females having age ranges from 6–11 years. From registration list, simple random sampling method was employed to select students from each section using a table of random numbers and when the selected student was absent, the student before or after the indicated one was sampled for replacement.

Students who were under taking anti parasitic drugs during the data collection date or refused in participation were excluded from the study.

Single stool sample was collected from each participant by sterilized stool cup. Three stool slides smears were processed for Wet mount, FEC and Kato Katz techniques from each sample and the slides were examined microscopically. The performance of Wet mount, Kato-Katz and FEC techniques were evaluated in terms of “Gold” standard. The Wet mount preparation and examination was performed as soon as the stool arrives in the laboratory. The FEC and Kato Katz techniques were processed depending on the test protocol and smeared on different slides. Finally, labeling of the slides was done with the students ID number.

### Diagnostic techniques

Wet Mount: In the Wet mount, fresh stool samples (approximately 2 mg of stool) were put on a slide with wooden applicator, emulsified with a drop of physiological saline (0.85 %) for diarrheic and semi solid or Iodine for formed stools, covered with cover slide and examined under microscope using first 10 × objectives and then 40 × objectives.

Formol ether concentration: This test was performed by mixing 0.5 g of faeces in 10 ml of normal saline in a glass container and mix thoroughly. Two layers of gauze were placed in a funnel and strain the contents into a 15 ml centrifuge tube. 2.5 ml of 10 % formaldehyde and 1 ml of ether was added. The solution was mixed well and centrifuge at 1000 revolution for 3 min. The supernatant was removed and the slides were prepared from the sediment. Two slides were prepared (one for saline and the other for iodine), covered with cover slide and finally examined with microscope.

Kato Katz technique: The Kato Katz quantitative cellophane thick smear method (Mahidol University, Thailand following the manufacturer’s instruction) was used. It was performed by transferring the sieved stool to the templates which delivers 41.7 mg of stool.

The stool was covered with cellophane which was previously immersed with malachite green. Identification of the ova or cyst of the parasites and quantification of the ova of *S. mansoni* and geo-helminths were done. Eggs counted per slide were multiplied by 24 to convert into number of eggs per gram (epg) of stool. The parasite load or intensity was defined as light (1–100 epg), moderate (101–400 epg) and heavy based (>400 epg) of stool for *S. mansoni* and geo-helminthes according to World Health Organization (WHO) guideline [13, 14].

In all cases, the results of the Wet mount slides were determined earlier to FEC and Kato Katz techniques with strict blinding. The independent readings of the Wet mount, FEC and Kato Katz slides by the experienced laboratory personnel were checked by the principal investigator. To avoid observer bias, two experienced laboratory personnel performed the microscopic examination of Wet mount, FEC and Kato Katz slide smears blindly and independently. The results of their observation were recorded for later comparison on separate sheets. A quality control was done by repeating all discordant results. The discordant smears were rechecked by the principal investigator using 100 × objectives.

### Data entry and analysis

Data were entered and analyzed using SPSS version 20. Since, there is no “Gold” standard method to detect intestinal parasites; the operational characteristics [sensitivity and negative predictive value (NPV)] of diagnostic tests were estimated using the combined results from the three methods as diagnostic “Gold” standard [15]. The combination of Wet mount, FEC and Kato-Katz tests was used as a “Gold” standard diagnostic test. Sensitivity, specificity, positive predictive value, NPVs and Kappa value of Wet mount, FEC and Kato-Katz techniques were calculated against the Gold standard.

### Ethical considerations

Ethical clearance was obtained from College of Medicine and Health Science ethical review committee, Bahir Dar University and from Tacharmachiho Woreda Education Office. Written informed consent was obtained from every study participant including the parents and the guardians of the children. Intestinal parasite positive cases were treated with antihelminthics and anti protozoals depending on the type of species identified.

## Results

Of 422 students participated in the study, 135 (32 %) and 287 (68 %) were males and females, respectively. The prevalence of intestinal parasites using single wet mount, FEC, and Kato-Katz thick smear techniques were 39.6, 50.7, and 74.6 %, respectively (Table 1). The detection rate when two techniques were used at a time was 77.5, 52.8 and 78.9 % for Kato Katz and FEC, Wet mount and FEC and Wet mount and Kato-Katz technique, respectively. The detection rate when all the three techniques were used together was 79.6 % (Table 1). Of the 422 study participants diagnosed 336 were found to be positive while the rest 86 were negative by the “Gold” standard method (Table 1). Furthermore, Kato-Katz detected 148 cases that were negative by a single wet mount (Table 1). The overall prevalence of *S. mansoni, Ascaris lumbricoides* and hookworm infections by the “Gold’ standard was 71.3, 2.8 and 7.1 %, respectively.

**Table 1:** The distribution of intestinal parasites identified in each diagnostic tests from May to June 2013

Types of methods	Result		
	No of examined (N)	Positive [N (%)]	Negative [N (%)]
Wet mount	422	167 (39.6)	255 (60.4)
FEC^a^	422	214 (50.7)	208 (49.3)
Kato Katz	422	315 (74.6)	107 (25.4)
Wet mount + FEC	422	223 (52.8)	199 (47.2
Wet mount + Kato Katz	422	333 (78.9)	89 (21.1)
FEC + Kato Katz	422	327 (77.5)	95 (22.5)
Wet mount + FEC + Kato Katz	422	336 (79.6)	86 (20.4)

^a^ *FEC* Formol ether concentration

### Performance of the test techniques

The sensitivity and NPV for Wet mount were 48.9 (95 % CI 48.8–49.1) and 33.7 (95 % CI 33.5–33.9), respectively (Table 2). The sensitivity and NPV of Kato-Katz were 93.7 % (95 % CI 93.6–93.7) and 80.4 % (95 % CI 80.1–80.6), respectively (Table 2). The FEC detected five negative samples that were positive by the Gold standard, indicating 48.9 % (95 % CI 48.8–49.1) and 33.7 % (95 % CI 33.5–33.9) sensitivity and NPV, respectively (Table 2).

**Table 2:** The performance of diagnostic techniques of intestinal parasitosis against the Gold standard method from May to June 2013

Techniques	Gold standard					
	Sensitivity (95 % CI)	Specificity (95 % CI)	PPV (95 % CI)^a^	NPV (95 % CI)^a^	P, χ^2^	Kappa value
Wet mount	48.9 (48.8–49.1)	94.5 (94.4–94.7)	97 (96.9–97.1)	33.7 (33.5–33.9)	0.00, 55.35	0.26
FEC^a^	63.1 (62.9–63.3)	94.5 (94.4–94.7)	97.7 (97.6–97.7)	41.3 (41.1–41.6)	0.00, 94.9	0.39
Kato Katz	93.7 (93.6–93.7)	94.5 (94.4–94.7)	98.4 (98.4–98.5)	80.4 (80.1–80.6)	0.00, 2.9	0.83

^a^ *PPV* positive predictive value; *NPV* negative predictive value *CI* confidence value *FEC* formol ether concentration

### Performances of Wet mount, Formol ether and Kato-Katz techniques by parasite types

Performances of Wet mount, Formol ether and Kato-Katz techniques by parasite types revealed that the specificity of Wet mount for *A. lumbricoides*, Hook worm species and *S. mansoni* were 99 (95 % CI 97.5–99.7), 99.2 (95 % CI 97.2–99.8) and 39 (95 % CI 33.7–44.7), respectively. The least diagnostic performance was observed in *S. mansoni* using Wet mount 39 (95 % CI 33.7–44.7) followed by FEC 50 (95 % CI 43.6–56.4) (Table 3).

**Table 3:** The ability of diagnostic techniques to detect geo-helminths and *S. mansoni* against the Gold standard from May to June 2013

Types of tests	Parasites identified					
	*S. mansoni* [N, %]	*A.lumbricoides* [N, %]	H. worm [N, %]	Total [N, %]	P, χ^2^	Kappa value
Wet mount	104 (31)	12 (3.8)	27 (8.0)	143 (42.6)	0.01, 72.2	0.29
FEC	166 (49.4)	12 (3.8)	30 (8.9)	208 (61.9)	0.01, 1.32	0.48
Kato Katz	292 (86.9)	12 (3.8)	30 (8.9)	334 (99.4)	0.01, 3.79	0.95

*H. worm* Hook worm species *FEC* Formol ether concentration

### Agreement in test results

The detection rate of Wet mount, FEC, and Kato-Katz technique for *S. mansoni* and geo-helminths were 99.4, 61.9 and 42.6 %, respectively. As compared to Wet mount and FEC, Kato-Katz test detected the highest number 292 (86.9 %) of *S. mansoni* infected cases (Table 3). The test agreement among Wet mount, FEC and Kato-Katz, for the diagnosis of *S. mansoni*, *A. lumbricoides* and hook worm species were poor (k = 0.29), moderate (k = 0.48) and excellent (k = 0.95), respectively (Table 3).

## Discussion

Although the combined result of the three methods used in this study is an approximation of the Gold standard, it is almost similar to the Kato- Katz technique. However, we used this combined result to compare the three techniques because of its superior detection capacity to Kato-Katz technique.

In this study, Kato Katz outperformed the other two methods, but the true values for sensitivity, specificity and diagnostic values are not known. In comparison to parasite recovery, our results confirmed that Kato Katz method (74.6 %) was high as compared to wet mount preparation (39.6 %) and FEC (50.7 %) methods. This result agrees favorably with other similar studies done previously [2, 16].

Taking the combined results of three techniques as a standard test for intestinal parasitic infection, the sensitivity and NPV of Kato-Katz was high. This was in agreement with the study done in Gondar [11].

The detection rate of intestinal parasite with wet mount (39.6 %) was lower than the FEC (50.7 %) in the present study. This result was in agreement with the study done in Nigeria [17].

In this study, the detection rate of *S. mansoni* by FEC and Kato Katz was higher than the wet mount. This was similar with previous study conducted in Gondar town [2].

As compared to direct wet mount and FEC techniques, the Kato-Katz revealed high sensitivity for the detection of *S. mansoni.* Similar previous result was found which showed Kato Katz test is best in *S. mansoni* detection [6]. This showed that the use of the Kato-Katz as a confirmatory test for *S. mansoni* will reduce the morbidity and mortality caused by this parasite by minimizing misdiagnosis.

Though, wet mount has low sensitivity to detect intestinal parasitosis, it is widely used as a routine means of diagnosis in Ethiopia. Our study finding showed that, direct wet mount exhibited very low sensitivity for the detection of *S. mansoni* and hookworm species as compared to the FEC and Kato-Katz techniques. This was in accordance with previous similar study [2]. This suggested that the use of direct wet mount alone as an intestinal parasitic infections identification is insufficient and may lead to false negative results.

The detection rate of hook worm species (9.1 %) was similar with FEC and Kato Katz techniques in the present study. Previous comparable results were obtained from 6.1 % for Kato Katz and 5.8 % for FEC [18] and 5.6 % in Kato Katz and 5.9 % in FEC [11] in hook worm detection.

Limitation of this study was that all the combined results of the three methods are highly influenced by parasite prevalence. Thus, the same method will have different values in different areas of prevalence.

## Conclusions

The present study revealed that the Kato Katz technique outperformed the other two methods but the true values for sensitivity, specificity and diagnostic values are not known. Moreover, it is labor intensive and not easily accessible. Hence, it is preferable to use FEC technique to complement the Wet mount test. Furthermore, Ministry of Health should work in collaboration with Universities to evaluate the diagnostic performance of Kato-Katz, FEC and Wet mount methods to use the better method.

## Abbreviations

FEC: formol ether concentration
WHO: World Health Organization
NPV: negative predictive value
